# Sudan's healthcare crisis: The struggle of kidney patients amidst conflict

**DOI:** 10.1002/hsr2.1980

**Published:** 2024-03-13

**Authors:** Emadeldin Hassan E. Konozy

**Affiliations:** ^1^ Department of Biotechnology Africa City of Technology (ACT) Khartoum Sudan; ^2^ Pharmaceutical Research and Development Centre, Faculty of Pharmacy Karary University Omdurman Sudan; ^3^ Biomedical and Clinical Research Centre Cape Coast University Cape Coast Ghana

**Keywords:** conflicts, healthcare struggles, humanitarian response, kidney dialysis, Sudan crisis

## Abstract

**Background and Aim:**

Conflicts, akin to other crises, disrupt lives and healthcare infrastructure, disproportionately affecting vulnerable individuals. The ongoing Sudanese conflict, initiated on April 15, 2023, between the Sudanese Armed Forces (SAF) and the Rapid Support Force (RSF), triggers significant population displacement, healthcare facility closures, and a scarcity of medical resources. Amid the intense conflict in Khartoum, reports of deceased individuals in the streets heighten concerns about public health, emphasizing the gravity of the situation. This crisis compounds the challenges faced by Sudan's already fragile healthcare system, impacting over 5 million displaced individuals, including those reliant on life‐saving hemodialysis sessions. This commentary aims to illuminate the challenges confronting kidney dialysis patients in war‐torn Sudan, emphasizing the impact on the Al‐Goled hemodialysis center.

**Methodology:**

This commentary relies on reports from the American Society of Nephrology (ASN), European Renal Association (ERA), and The International Society of Nephrology (ISN), along with recent journal articles discussing the consequences of ongoing conflicts. Personal observations in Al‐Goled contribute to the insights, and data from the Al‐Goled hemodialysis center in Sudan illuminate the struggles faced by kidney dialysis patients during the conflict, presented as a case study.

**Results:**

Kidney dialysis patients, crucial for regular hemodialysis sessions, confront severe challenges due to the overwhelming conflict. With an estimated 8000 kidney failure patients in Sudan, disruptions in healthcare services, targeted attacks on medical staff, and a shortage of resources exacerbate their plight. The Al‐Goled hemodialysis center, initially designed for 30 daily sessions, experiences a surge in demand, accommodating 85 patients daily—an alarming 183% increase. Limited resources, machine malfunctions, and a shortage of medications contribute to the loss of 13 patients' lives.

**Conclusions:**

The conflict in Sudan disproportionately impacts kidney dialysis patients, causing disruptions in essential healthcare services. The surge in demand at the Al‐Goled center underscores the broader impact on healthcare institutions nationwide. Local efforts to source medical supplies face bureaucratic hurdles and complex exportation procedures, impeding support for kidney patients. Patients, once receiving 3 weekly sessions, now struggle to secure even one, jeopardizing their well‐being. Urgent international intervention is needed to cease the conflict and ensure the safety of healthcare facilities, especially for vulnerable populations like kidney dialysis patients.

## 1 INTRODUCTION

Conflicts, akin to other disasters, significantly disrupt the lives of individuals with illnesses by damaging critical infrastructure and hindering their ability to reach life‐saving treatments.^1^ Patients in affected areas, including displaced individuals, confront increased risks due to potential inadequate care, resulting in elevated morbidity and mortality rates. The ongoing conflict between the Sudanese Armed Forces (SAF) and the paramilitary Rapid Support Force (RSF) since April 15, 2023, has triggered substantial population displacement, leading to the closure of multiple healthcare facilities and a shortage of essential medical resources and personnel.^2^, ^3^

Amidst the intense conflict in Khartoum, where a significant portion of the country's population resides, distressing reports have emerged of deceased civilians and soldiers abandoned in the streets, raising concerns about infection outbreaks and public health.^4^ Already, documented cases of cholera and dengue fever outbreaks in cities adjacent to Khartoum underscore the gravity of the situation. These compounding factors further strain Sudan's already fragile healthcare system.^5^

Families are separated, and countless lives are disrupted, leading to a profound humanitarian crisis affecting all aspects of Sudanese life.^6^, ^7^, ^8^ Additionally, the intense conflict in the capital has forced many government and private medical facilities to close, either due to crossfire, shelling, or the tragic loss of medical staff. Operational facilities are struggling to provide healthcare services. According to the World Health Organization's Situation Report No. 4 dated December 15, 2023, titled “Sudan Health Emergency,” there have been 60 verified attacks on healthcare facilities since April 15, 2023, resulting in 34 fatalities and 38 injuries.^9^ The scarcity of resources, especially essential medical supplies, compounds an already dire crisis.^4^ In this complex scenario, the lives of many individuals have been profoundly affected. However, for those who rely on regular dialysis treatments, displacement presents an exceptionally daunting obstacle. Kidney patients are among the most vulnerable casualties of the war, facing increased risks due to the specialized nature of kidney care and the scarcity of facilities providing such treatment.^10^ From this perspective, it is crucial to thoroughly explore the crisis that individuals undergoing kidney dialysis are facing.

## 2 KIDNEY DIALYSIS PATIENTS' STRUGGLE IN WARTIME SUDAN

Kidney dialysis patients, who rely on regular hemodialysis sessions to stay alive, highlight the profound human toll exacted by this crisis. Before the onset of the war, in Sudan, it was estimated that there were 8000 kidney failure patients in the country, all of whom required 70,000 dialysis sessions per month to stay alive.^11^ However, amidst the chaos of the war, kidney dialysis patients found themselves in an increasingly desperate situation. The entire healthcare system was overwhelmed, and medical professionals were forced to make heart‐wrenching choices, leaving some patients without the care they urgently needed. Militia groups specifically targeted and killed doctors, nurses, and other medical staff, fostering an environment of fear and mistrust. A joint report from the American Society of Nephrology (ASN), European Renal Association (ERA), and The International Society of Nephrology (ISN) titled “Kidney Organizations Appeal for Kidney Health for All War Victims” expresses deep concern and sorrow for kidney impairment patients facing incredible challenges due to the ongoing conflict in Sudan.^12^ The report estimates that around 8000 people in Sudan depend on hemodialysis for survival, while approximately 4500 are kidney transplants.^13^ The supplies required for hemodialysis sessions are running perilously low, with procurement and delivery presenting significant challenges, particularly in war‐affected regions. In Khartoum, there are only a limited number of operational dialysis centers located in relatively safer areas. However, persistent and, at times, months‐long power failures have presented significant obstacles, making it extremely challenging for these centers to function effectively. Some of these centers' management attempted to address this issue by bringing in electricity generators to facilitate patients' routine sessions, but acquiring petrol for these generators is yet another problem they face. Moreover, the water infrastructure has been compromised, and these centers now have to purchase water for a fee. This water is sourced, sometimes, directly from the Nile River without adequate attention to water sanitation precautions. The situation has reached a grim point where kidney dialysis patients are losing their lives, and their bodies are left exposed to decompose, as reported by Reuters.^14^

## 3 HOPE AND HARDSHIPS: THE AL‐GOLED HEMODIALYSIS CENTER

During the upheaval of the conflict, my family and I sought sanctuary in the northern Sudanese town called Al‐Goled. This quaint town holds profound familial significance, being the birthplace and upbringing locale of my ancestors, including my parents. Hence, our deliberate choice to find refuge in Al‐Goled aimed to distance ourselves from the unpredictable capital of Khartoum. Being present in Al‐Goled provided me with the opportunity to witness firsthand the hardships faced by kidney hemodialysis patients. In this town, a modest hemodialysis center stands as a symbol of compassion and a testament to the resilience of community solidarity. Gifted by a local philanthropist, this center has assumed a mission of paramount importance amid challenging circumstances. Originally designed to manage around 30 dialysis sessions per day, the Al‐Goled hemodialysis center served not only patients from Al‐Goled itself but also those from nearby cities, villages, and towns. It stands as a poignant portrayal of the unfolding tragedy in Khartoum, with repercussions extending beyond the city's healthcare system. The facility, equipped with 15 hemodialysis machines, was intended to accommodate 30 patients daily—15 during the morning shift and an equal number in the afternoon. However, the outbreak of conflict in Khartoum, leading to the closure of multiple centers, resulted in a sudden influx of patients to Al‐Goled. This abrupt surge elevated the daily patient load from 30 to 85, marking a substantial 183% increase. Managing this significant rise, compounded by a shortage of hemodialysis medications and solutions, along with the recurrent malfunctioning of the limited number of machines and the challenge of securing biomedical engineers for repairs, presented formidable challenges. Unfortunately, these awful circumstances contributed to the tragic loss of 13 patients' lives. Similar to many other centers nationwide, this center used to depend on Khartoum as its primary source for medical supplies and intravenous solutions. However, the ongoing conflict has disrupted their supply chain, causing it to nearly disappear. In response, the residents of Al‐Goled have established committees to mobilize local efforts and seek assistance from charitable individuals living abroad. Their objective is to procure medical supplies using their own resources and connections, with the goal of easing the suffering of numerous kidney patients who undergo dialysis sessions at the Al‐Goled hemodialysis center. Unfortunately, these earnest efforts have encountered numerous obstacles, primarily stemming from bureaucratic processes within government offices and complex exportation procedures. The consequences of this overflow are nothing short of heart‐wrenching. Patients who depend on these life‐saving dialysis sessions have seen their routines dramatically disrupted. Where they were once able to receive three sessions a week, they now find themselves struggling to secure just one. As I pen this perspective, even that single session remains uncertain and fragile due to the formidable obstacles presented by government precited procedures, which hinder the supply of crucial medical resources. Adhering to government protocols, government medical insurance encompasses all hemodialysis patients, enabling them to undergo weekly dialysis sessions at a reduced cost. However, patients bear the responsibility of acquiring specific medications and solutions, such as phosphate binders and antihypertensives, directly from private pharmacies. On the flip side, the government provides the remaining chemicals, drugs, and related materials, including eprix, acid concentrate, bicarbonate, and dialyzers. Unfortunately, the ongoing conflict has led to a scarcity of these supplies, posing a challenge in consistently obtaining them from the hospital and resulting in a reduction in dialysis sessions.

## 4 CONCLUSION REMARKS

The plight of kidney dialysis patients in war‐ravaged Sudan underscores the urgent need for international intervention. The conflict has created severe disruptions in healthcare services, leading to a surge in demand at the Al‐Goled center and contributing to the loss of lives. Local efforts to address the scarcity of medical supplies face bureaucratic obstacles, hindering support for vulnerable patients who are now struggling to secure even 1 weekly session. Immediate action from the international community is imperative to cease the conflict, ensure the safety of healthcare facilities, and provide essential medical resources. Saving the lives of those dependent on hemodialysis requires a collaborative effort to bring about a ceasefire, facilitating the delivery of crucial supplies and enabling uninterrupted access to life‐saving treatments. The international community's involvement is not only a moral imperative but a vital step towards alleviating the suffering of kidney dialysis patients and preventing further escalation of this humanitarian crisis.

## AUTHOR CONTRIBUTIONS

**Emadeldin Hassan E. Konozy**: Conceptualization; investigation; writing—original draft; writing—review and editing; project administration; data curation.

## TRANSPARENCY STATEMENT

The lead author Emadeldin Hassan E. Konozy affirms that this manuscript is an honest, accurate, and transparent account of the study being reported; that no important aspects of the study have been omitted; and that any discrepancies from the study as planned (and, if relevant, registered) have been explained.

## Data Availability

The data that support the findings of this study are available from the corresponding author upon reasonable request.

## References

[1] Vanholder R , Gallego D , Sever MS . Wars and kidney patients: a statement by the european kidney health alliance related to the Russian‐Ukrainian conflict. J Nephrol. 2022;35(2):377‐380. 35246798 10.1007/s40620-022-01301-4 PMC8897116

[2] Konozy EHE . Sudan's devastating war: unravelling its multifaceted impact. Med Confl Surviv. 2023:1‐5. https://www.tandfonline.com/doi/full/10.1080/13623699.2023.2299736 10.1080/13623699.2023.2299736 38151893

[3] Dafallah A , Elmahi O , Ibrahim ME , Elsheikh RE , Blanchet K . Destruction, disruption and disaster: Sudan's health system amidst armed conflict. Confl Health. 2023;17(1):43. 37752590 10.1186/s13031-023-00542-9 PMC10523736

[4] Hemmeda L , Ahmed AS , Omer M . Sudan's armed rivalry: a comment on the vulnerable healthcare system catastrophe. Health Sci Rep. 2023;6(8):e1517. 37621384 10.1002/hsr2.1517 PMC10444969

[5] Nashwan AJ , Osman SH , Mohamedahmed LA . Violence in Sudan: a looming public health disaster. Cureus. 2023;15(6):40343. 10.7759/cureus.40343 PMC10338988 37456400

[6] IOM's Global Crisis Response Platform. *Internal Displacement in Sudan Nearly Doubles Since Onset of Conflict*. IOM; September 5, 2023.

[7] World Health Organization. *Sudan Health Emergency*. Situation Report No. 3, September 30, 2023.

[8] Office for the Coordination of Humanitarian Affairs. *Number of Children Displaced Across Sudan Now Highest in the World*. Office for the Coordination of Humanitarian Affairs; 2023.

[9] World Health Organization . *Sudan health emergency: situation* (Report No. 4). Retrieved from https://www.emro.who.int/images/stories/sudan/WHO-Sudan-conflict-situation-report-15-December_2023.pdf

[10] Novakivskyy V , Shurduk R , Grin I , et al. War in Ukraine and dialysis treatment: human suffering and organizational challenges. Clin Kidney J. 2023;16(4):676‐683. 37007698 10.1093/ckj/sfad003 PMC10061431

[11] Middle East Monitor. *Kidney Patients Struggle to Get Dialysis Sessions Amidst Sudan War*. Middle East Monitor; 2023.

[12] International Society of Nephrology. *Support the Kidney Care Community in Sudan to Overcome Critical Challenges Caused by the Conflict*. International Society of Nephrology; 2023.

[13] Hassan HA , Hafez MH , Luyckx VA , Tuğlular S , Abu‐Alfa AK . Kidney failure in Sudan: thousands of lives at risk. Lancet. 2023;402(10402):607. 10.1016/S0140-6736(23)01370-3 37572679

[14] Makary A. Sudan's war exacts deadly toll on dialysis patients, leaves bodies rotting. *Reuters*. June 15, 2023.

